# On the Use of Deep Active Semi-Supervised Learning for Fast Rendering in Global Illumination

**DOI:** 10.3390/jimaging6090091

**Published:** 2020-09-08

**Authors:** Ibtissam Constantin, Joseph Constantin, André Bigand

**Affiliations:** 1 LaRRIS, Faculty of Sciences, Lebanese University, Fanar, BP 90656 Jdeidet, Lebanon; ibtissamconstantin@ul.edu.lb (I.C.); cjosephconstantin@gmail.com (J.C.); 2 LISIC, University of Littoral Côte d’Opale (ULCO), Calais, BP 719, 62228 Calais CEDEX, France

**Keywords:** active semi-supervised learning, convolution neural network, stochastic noise detection, rendering algorithm

## Abstract

Convolution neural networks usually require large labeled data-sets to construct accurate models. However, in many real-world scenarios, such as global illumination, labeling data are a time-consuming and costly human intelligent task. Semi-supervised learning methods leverage this issue by making use of a small labeled data-set and a larger set of unlabeled data. In this paper, our contributions focus on the development of a robust algorithm that combines active and deep semi-supervised convolution neural network to reduce labeling workload and to accelerate convergence in case of real-time global illumination. While the theoretical concepts of photo-realistic rendering are well understood, the increased need for the delivery of highly dynamic interactive content in vast virtual environments has increased recently. Particularly, the quality measure of computer-generated images is of great importance. The experiments are conducted on global illumination scenes which contain diverse distortions. Compared with human psycho-visual thresholds, the good consistency between these thresholds and the learning models quality measures can been seen. A comparison has also been made with SVM and other state-of-the-art deep learning models. We do transfer learning by running the convolution base of these models over our image set. Then, we use the output features of the convolution base as input to retrain the parameters of the fully connected layer. The obtained results show that our proposed method provides promising efficiency in terms of precision, time complexity, and optimal architecture.

## 1. Introduction

In computer graphics, the goal of photo-realistic rendering is to create convincing images, given the description of a world. This process involves different areas like video production [1] and manufacturing [2]. Path-Tracing can be seen as a random walk that makes it possible to estimate the value of a pixel by randomly sampling many paths and computing the average contribution based on Monte Carlo path tracing algorithm [3]. Then, producing photo-realistic images requires computing a complex multidimensional integral of the scene function at every pixel of the image. Due to the high cost of tracing millions of paths, the cost of computing one image is excessive for computer graphics applications. Thus, a challenging problem is to dramatically reduce the computation time in order to provide a practical real-time global illumination.

Different models have been developed to simulate the behavior of the human visual system. Some of these models are based on full reference quality metrics in order to detect the difference between two images while the others use reduced reference machine learning algorithms. In case of quality metrics, the Visible Differences Predictor model was proposed to predict the probability of detecting the difference between two images [4] while the Sarnoff Visual Discrimination Model was based on a set of complex sub-models that simulate several aspects of the human visual system [5]. However, these models are complex and incomplete due to the human system’s complexity. Hence, they need the reference image which is the visually converged one and they require long computation times and are difficult to parametrize [6]. Considering the fact that in path tracing algorithm, the reference image is not initially available like in classic image processing methods, we try in this paper to apply reduced reference quality assessment techniques by searching noise attributes using deep architecture of convolution neural networks.

Machine learning algorithms have provided models for accelerating global illumination rendering by detecting stochastic noise [7,8,9]. Some of these models used support vector machine (SVM) and relevance vector machine (RVM) to train the network with labeled data images [8,9]. Another model used a deep spike neural network with a clustering dynamic algorithm for the training images [7]. The main idea behind these techniques is to detect the increasing quality of images by estimating decreasing noise level using noise attributes. However, the proposed models require large amounts of carefully chosen labeled images to tune their parameters in order to give a good precision on the testing scenes.

One of the main assets of deep learning algorithms over other machine learning algorithms is their great modeling capacity, which allows them to handle complex high-dimensional data-sets through feature representations. Convolution neural networks require large labeled data-sets to construct accurate models; however, in many real-world scenarios, labeling data are a time-consuming and costly human intelligent task. Active and semi-supervised learning algorithms are invented to improve the classification accuracy and to reduce labeling workload using both labeled and unlabeled data. An active learning algorithm takes the images that have the lowest confidence as the most informative ones [10,11]. It selects such images and asks the expert for its label. Active learning needs human involvement and aims at selecting the most useful images for training. It can improve the model’s performance and can accelerate the convergence speed. The semi-supervised learning method selects the images that have the highest confidence and adds the predicted label by the machine itself without any human involvement [12]. However, when the initial model is very weak, many labels would be wrongly predicted, and thus it would introduce mistakes into the training set.

In active learning, uncertain and erroneous portion of the training images are required to be queried, annotated manually with minimum human cost, and added to the training set to get the highest gain in classification accuracy. In the semi-supervised learning, the classifier is constructed beginning with a few labeled training images. A portion of the unlabeled training images that are not selected during active learning are labeled using the current classifier, and the most confident images among the predicted labeled images are added to the training set, repeatedly, until convergence. It can be seen that there is a very good complementarity between active and semi-supervised learning. Thus, we integrate them in global illumination rendering in order to reduce the size of the large labeled data-sets that are needed to efficiently train convolution neural networks and to improve model performance.

In this paper, we present an active semi-supervised learning algorithm based on deeply embedded feature representations by alternatively using labeled and unlabeled data images for real-time and interactive global illumination. We use a deep architecture which is a powerful machine learning technique from the field of deep learning in order to extract a rich feature representations for a wide range of images [13]. The role of deep learning is to create higher level noise prediction through the use of multiple layers of nonlinear operations. In order to further reduce manual labeling workload, the semi-supervised learning algorithm is designed to make full use of those unlabeled images that are not queried by active learning [14,15]. The model uses semi-supervised learning to select class central sub-images that are not selected during active learning based on different layers for features extraction, concatenation, and prediction [14,16]. It has also been shown that semi-supervised learning overcomes the limitations of supervised learning in many different applications [14,17]. However, to our knowledge, its performance was not well investigated in the domain of stochastic noise detection for rendering algorithms using deep convolution neural networks. The contributions of this paper are summarized in the following:

- We used active semi-supervised learning for efficient stochastic noise detection in global illumination rendering algorithms.
- We combined active and semi-supervised learning to minimize the effect of imbalanced image classification in order to model the capability of the deep feature distribution for efficient noise detection.
- We extensively evaluated our algorithm on different global illumination scenes with different resolutions containing diffuse and specular surfaces. The proposed algorithm demonstrates outstanding performance compared with state-of-the-art algorithms applied for accelerating global illumination rendering.
- We made a comparative study based on memory space and computation time. We showed that our model is computational efficient for real world applications because it yielded a small questioning time on a block of images.

The motivation behind this work is to perform a rapid calculation of the illumination in the rendered images. The convolution neural network is proposed to handle the data, but due to the typical training of the deep neural network, we propose a semi-supervised training algorithm, which improves the performance and makes the approach suitable for real-time applications. One key issue for most deep learning algorithms is that they need large amounts of labeled images to train the model. Since manual labeling is time-consuming, we have proposed to combine active learning and semi-supervised learning to reduce manual labeling workload. Realizing that active learning algorithm is only interested in images that are more likely to be on the class boundary, while ignoring the usage of the rest large amounts of unlabeled images, this paper designs a semi-supervised learning algorithm to make full use of the rest of the non-queried images. The proposed active semi-supervised algorithm uses active learning to select class boundary images, and semi-supervised learning to select class central images. By adding class central images, we are believed to better describe the class distribution, and to help active learning to find the boundary images more precisely.

The paper is structured as follows: Section 2 gives a discussion on related work on active semi-supervised learning, Section 3 describes how to design the image quality database, and Section 4 describes the architecture of the models and the learning methodologies. Section 5 shows the experimental results. Section 6 gives a discussion summarizing the advantages of the proposed approach and the outcomes and findings of the experimental analysis. Finally, the paper is summarized with some conclusions in Section 7.

## 2. Related Work on Active Semi-Supervised Learning

One of the main drawbacks using deep learning is about the necessity of a huge learning database. A large amount of labeled computer-generated images is expensive to acquire, but unlabeled images are easy to generate. Thus, the idea of active learning (AL) [18] and semi-supervised learning [19] to improve our classification task using unlabeled images is investigated. These two iterative procedures (AL and SSL) make it possible to improve machine learning in a lot of applications. At each iteration, AL selects the image that has the lowest confidence as the most informative one and asks the human observer for its label (noisy or not). At the same time, SSL selects the images that have the highest confidence and adds the predicted labels by the machine without any human investigation (HVS that takes about one hour per image). Thus, this procedure is very efficient in case of computer-generation of images.

The scientific literature on semi-supervised learning (SSL) is growing rapidly, showing significant performance gains in recent years. A semi-supervised feature extraction algorithm has been developed for pattern categorization [20]. However, this algorithm was tested only for classification on benchmark databases without considering experimental data for real-life applications. The performance of the inductive rules family classifiers has been improved by presenting a new technique for the presentation order of the training samples, which combines a clustering method with a density measure function [21]. The main drawback of this approach is that the convergence to a good result could be quite time-consuming especially when the training set contains thousands of samples. Thus, it cannot be applied in case of global illumination because the learning algorithm should select pertinent samples from a set containing a huge number of unlabeled images. Recently, ref. [22] proposed to use manifold embedding with deep learning architecture to improve the results using the structural information of the data.

A group of semi-supervised methods consists of three different models trained on the same dataset using bootstrap sampling. After a supervised training of every model, an unlabeled data point is added to the training set of one model only if the other two models agree on its label [23]. Another type of semi-supervised methods can be referred to as self supervised methods. These algorithms learn to exploit robustness to stochastic perturbations caused by noise or randomness in data augmentation [24,25,26]. This idea has been recently applied by [27] for image set classification with success. The idea of integrating active method along with semi-supervised learning was also introduced in the scientific literature. An active based semi-supervised SVM (SVM, support vector machine) learning algorithm was proposed in [28,29]. These algorithms used active learning to improve the performance of the initial classifier, and then the semi-supervised learning algorithm was designed to assign labels to the remaining unlabeled samples. Other research papers combined active with semi-supervised learning for image compression, speech recognition, and multi-task learning [30,31,32]. A very competitive class of semi-supervised learning algorithms was proposed to exploit robustness in case of stochastic noise [33]. However, the proposed models take a full dimension of the image as input without using a deep convolution neural network (CNN), which is shown to be successful. Indeed, CNNs can extract appropriate noise features while jointly performing discrimination. Moreover, the performance of the developed algorithms was not investigated in case of scenes with different resolutions and by using different rendering scenarios. The goal of this paper is to create a new approach based on deep active semi-supervised learning to automatically allow a stopping criterion when a perceptive convergence is reached in global illumination methods. We show the advantage of this model in terms of parameters number and high precision by comparing it with SVM.

## 3. Design of the Image Quality Database

Different Image Quality databases are available to test the performance of the learning algorithms with respect to the human visual thresholds. However, scenes captured using camera devices are usually afflicted by mixture of multiple distortions that do not modelize synthetic distortions found in existing databases well [34,35]. The model is built on data corresponding to images of globally illuminated scenes. The path tracing algorithm was used in order to reduce noise. This algorithm generates stochastic paths from the camera to the 3D scene. For each intersection of a path with the surface, a direction of reflection or refraction is randomly extracted. The luminance at a point *x* in direction *w* is defined by [36]:

(1) $$L\left(x,w\right)=L_{e}\left(x,w\right)+\int_{S}V\left(y,x\right)f_{r}\left(w_{yx},x,w\right)L_{in}\left(y,x\right)G\left(y,x\right)dA\left(y\right)$$

where *S* is the scene surface, $L_{e}$ is the emitted luminance, *V* is the mutual visibility, $f_{r}$ is the bidirectional scattering distribution function, $w_{yx}$ is the direction from *y* to *x*, $L_{in}$ is the incidence luminance, $G\left(y,x\right)=\cos\theta_{y}\cos\theta_{x}/{∥y-x∥}^{2}$, θ is the direction between incident and normal directions, and *A* is the area measure.

**Figure 1:**
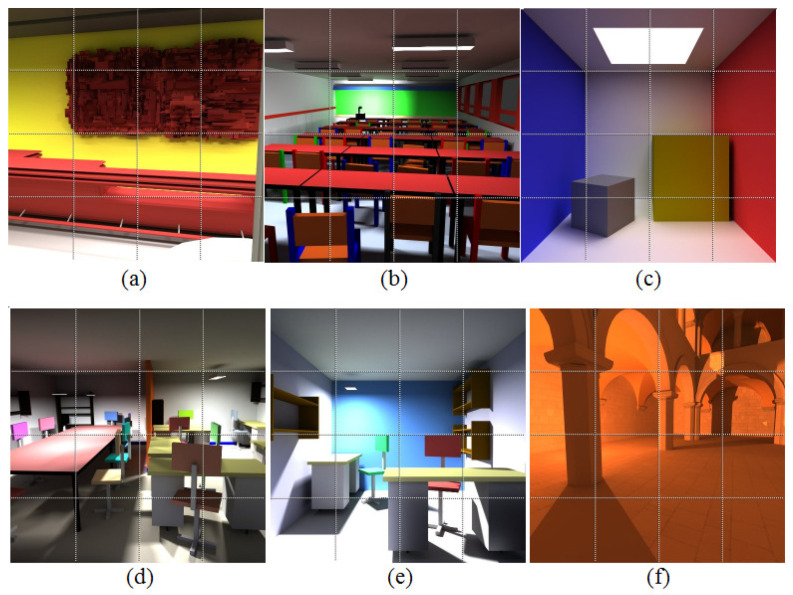
Rendering results for the sub-images of the scenes with 512 × 512 resolution. The scenes with 512 × 512 resolution (**a**–**f**) consist of diffuse surfaces with complex geometry. They contain objects with different material properties presenting rich and complicated shading variations under different lighting conditions.

**Figure 2:**
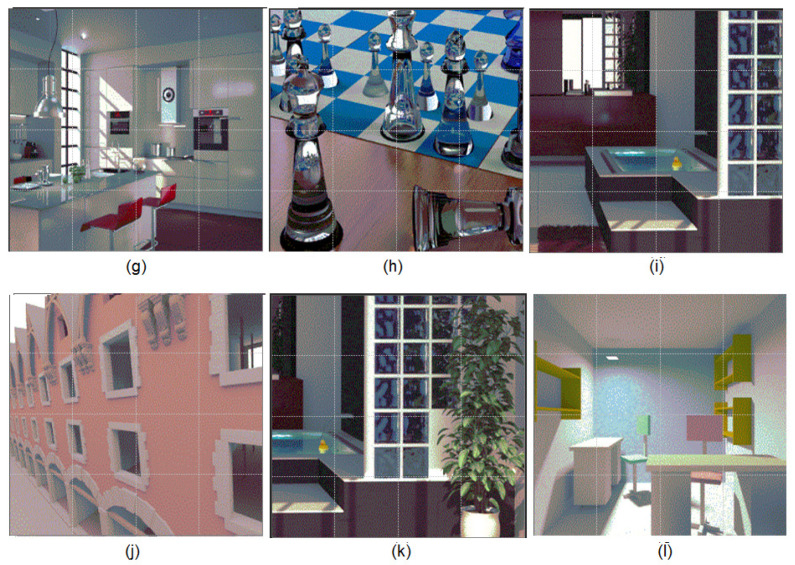
Rendering results for the sub-images of the scenes with 800 × 800 resolution. The scenes with 800 × 800 resolution (**g**–**l**) are used to illustrate view-dependent indirect illumination effects caused by strong inter-reflections between the diffuse and specular surfaces.

The luminance of a pixel is evaluated recursively based on a Monte Carlo technique [37]. For each pixel, the final luminance is the average of the contributions of all generated paths. We can then compute several images from the same point of view by adding equally, between two successive images, a certain number of new paths for each pixel. For each scene, several images were obtained, the first one being very noisy and the last one being the reference image. In order to get experimental data about noise perception, pairs of images were presented to the observer. One of these images, called reference image, was computed with a high number of paths per pixel. The second image, the so-called test image, was chosen from a stack of images arranged from very noisy ones above to converged ones below. During the experiments, the observer was asked to modify the quality of the noisy image by pointing to the areas where the differences were perceived between the current image and its reference one. Each operation then entailed the selection and display of the corresponding next level sub-image by reducing visually the noise in this image’s sub-part. This operation was done until the observer considered that the two images are visually identical. This operation is reversible meaning that an observer is able to go down or up into the images’ stack. Note that all the observers worked in the same conditions, the same display with identical luminance tuning, and the same illumination conditions. The results were recorded for 33 different observers and the average number of paths required for each sub-image to be perceived as identical to the reference one by 95% of the observers were computed. We tested the performances of our algorithms on global illumination scenes with 512×512 and 800×800 resolutions. For the classic scenes with 512×512 resolution, the images were cut into sixteen non-overlapping blocks of sub-images of size 128×128 pixels (Figure 1). The maximum number of paths per pixel was set to 10,100 in order to obtain non-distorted copies of the sub-images. The sub-images were computed by adding 101 paths for each pixel between two successive sub-images using the path tracing algorithm [38]. The scenes with 800×800 resolution were computed for diffuse and specular rendering (Figure 2). The human vision system (HVS) thresholds for each sub-image were then tested. In this case, the images were cut into 16 non-overlapping sub-images of size 200×200 pixels. The number of paths per pixel between two successive sub-images and the largest number of paths per pixel were set differently for each scene in order to test the performance of the deep active semi-supervised learning algorithm using different scenarios (Table 1).

**Figure 3:**
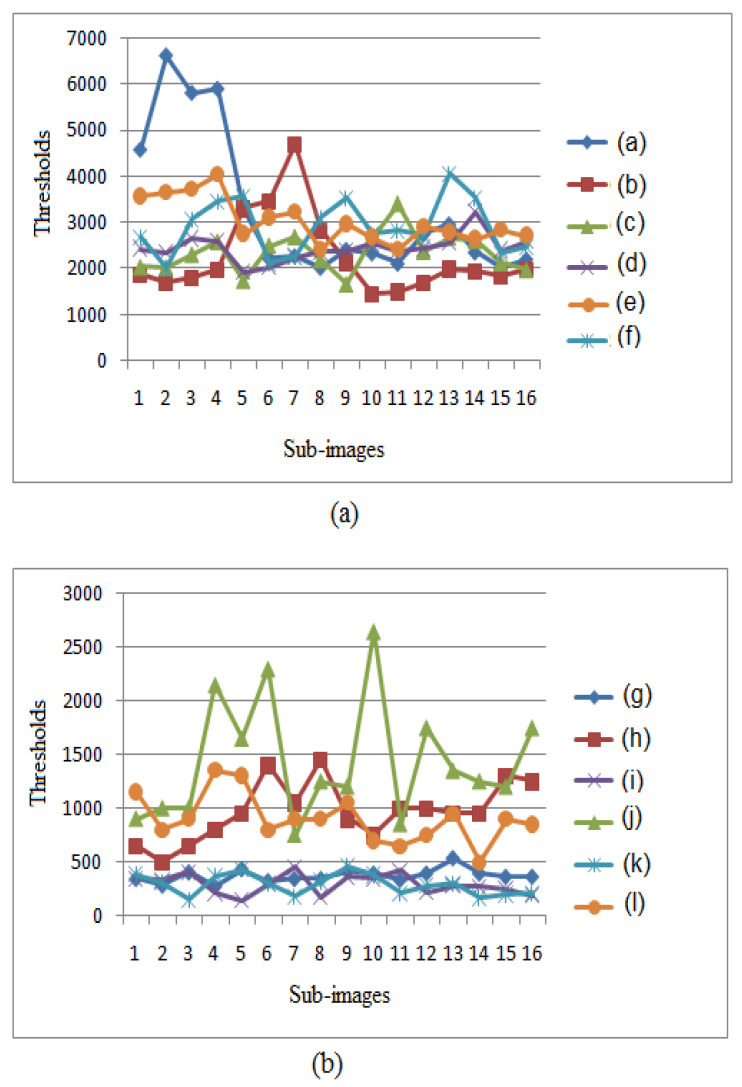
(**a**) desired thresholds for the sub-images of the scenes with resolutions 512×512 (**a**) and 800×800 (**b**).

The labeling process selected sub-images computed using diffuse and specular rendering and asked the observers for their qualities [8]. The average number of paths required for each block of sub-images to be perceived as identical to the reference one is shown in Figure 3.

## 4. The Proposed Method

### 4.1. Architecture of the Convolution Neural Network

Convolution neural networks are usually composed of several processing layers, each layer involving linear as well as nonlinear operators that are jointly learned in an end-to-end manner to solve a particular task [39,40]. In practice, very few people train an entire convolution neural network from scratch because it is relatively rare to have a dataset of sufficient size. Instead, it is common to take a convolution neural network that is pre-trained on a different large dataset and then use it either as a feature extractor or as an initialization for a further learning process.

**Figure 4:**
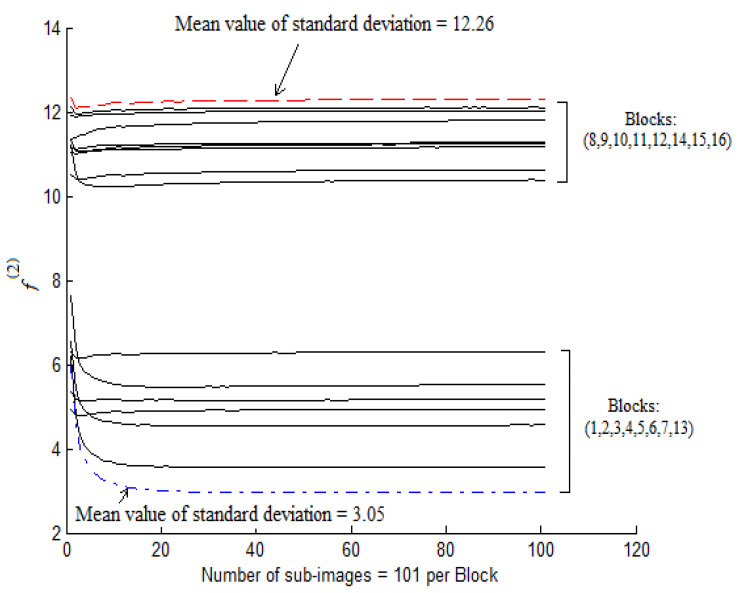
Standard deviation variation per block.

Feature extraction consists of using the representations learned by a previous network to extract interesting features from new images. These features are then introduced to a new prediction model which is trained from scratch. In order to extract the noise features, we took the convolution base of a previously trained network [7], ran the data on it, and trained a new prediction model on top of the output. The network was designed using twelve convolution layers of depth and spread equal to one [41,42,43] (see Table 2). Next, we inserted a thirteen layer which applies a one stage 2D wavelet decomposition on the input sub-image in order to extract noise from the wavelet coefficients in the high frequency sub-bands [44]. The sub-image noise was estimated as a pixel subtraction between the current sub-image and the sub-image computed by each layer. The mean and the standard deviation pooling were applied to the thirteen activation layers. We performed such image analysis employing statistical noise features because these features can effectively distinguish digital images from their tampered versions [45,46]. We plot the values of the standard deviation versus each sub-image per block (Figure 4). It is shown that these features are important to the learning models because their values monotonically change until they reach stable thresholds when the sub-images are not affected by noise.

**Figure 5:**
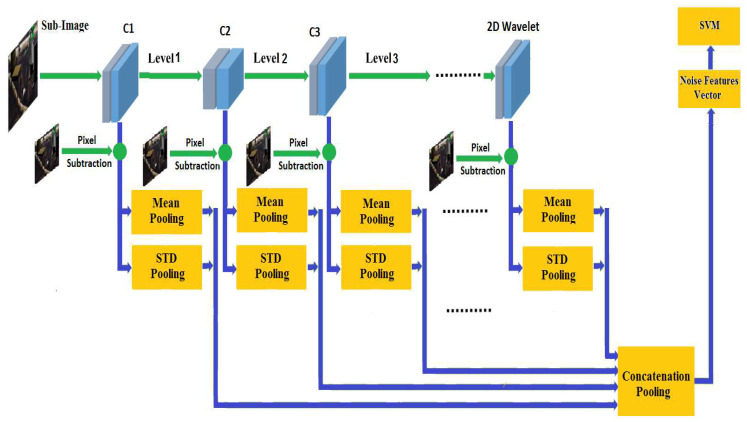
Architecture of the convolution neural network.

Next, the feature concatenation pooling was performed by grouping the version feature vectors in a single feature vector in order to obtain a total of 26 noise feature vector used as input to the model. The architecture of the convolution neural network is shown in Figure 5.

**Figure 6:**
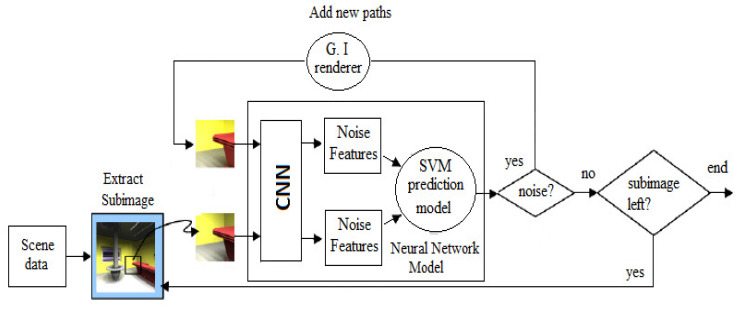
A schematic representation of the Path Tracing progressive algorithm including noise detection for each sub-image. At each iteration, new paths are added to the un-converged sub-images. Then, the model is tested if each new sampled sub-image is still noisy or not. According to the models answer, we decide then to add new paths or to stop computation for the corresponding sub-image as it is supposed to be visually converged.

The input to the prediction model was obtained by computing the difference between the noise features of a quick ray traced sub-image of the scene and the current one. Finally, we ran the convolution neural network over our data-set, thus recorded its output and then used this data as input to train a support vector machine model called DSVM and an active semi-supervised learning model called ${\mathrm{DSVM}}_{\mathrm{AS}}$ in order to predict the quality of the global illumination sub-images (Figure 6).

Next, these models were compared with the standard support vector machine (SVM) and the active semi-supervised SVM called ${\mathrm{SVM}}_{\mathrm{AS}}$ by considering the full luminance component of Lab color sub-images (*L*) as input and applying to *L* four different denoising algorithms: Linear filtering with averaging and Gaussian filters, median filters and adaptive Wiener filters. Each sub-image was also denoised using Wavelet analysis [33]. A comparison has also been made with the pre-trained VGG19 deep convolution neural network in order to show the efficiency of our work. The output of all models contained only one neuron which gave the value −1 for noisy sub-images and the value +1 for low-noisy sub-images.

### 4.2. Active Semi-Supervised Learning of Noise Features

#### 4.2.1. Active Learning

Active learning has been developed to improve learning ability in complex environments [47] such as our task. Active learning is a kind of semi-supervised learning algorithm that selects the most informative images to be labeled by an expert. In active learning, an uncertain portion of the training image set is required to be queried, annotated by an expert with minimum human cost and added to the training images set to get the highest gain in prediction accuracy. Most active learning methods select only a single image at each iteration. In active learning batch mode, a batch of images are selected at each step, and instead of updating the model at each iteration, the update is performed once at each batch iteration [48]. It is shown that the images closest to the current hyperplane are informative so they can shrink the size of the version space (the version space is defined in [49]), and, in this case, active learning can accelerate the convergence of the model when the version space is symmetric. Such images are more likely to be on the class boundary; that is to say, they have a high probability to accelerate the convergence of the model.

The active learning algorithm tries to query the sub-images that are the closest to the current classification hyperplane for each iteration. However, the prerequisite that the version space should be symmetric cannot be satisfied in many real-life situations. Moreover, when the labeled training set is small, it is difficult to describe the spatial distribution of the pattern classes. As a result, the sub-images closest to the current classification hyperplane may be less likely to be on the class boundary, and then selecting them is not the best choice. To solve this problem, we try in this paper to use semi-supervised learning in order to add some sub-images that can better describe the class distribution into the labeled training set, then the class boundary would be more clear, and it could be very beneficial for active learning to precisely select the class boundary samples. We take the class central sub-images as the ones that can better describe the class distribution.

#### 4.2.2. Deep Semi-Supervised Learning

In this paper, we adopt a flexible strategy by integrating the uncertainty from the concept of active learning and the confidence criterion from that of semi-supervised learning (see [50] for more details). Semi-supervised learning is widely used in deep learning frameworks [51], where the learning dataset is often a challenging task. The active semi-supervised learning algorithm initially trains the prediction model to extract noise features from a small set of confidently labeled sub-images, and it repeatedly retrains the prediction model by adding the batch of sub-images features selected by the active learning algorithm. Then, the confidence criterion of semi-supervised learning is applied to produce machine-labeled sub-images. Finally, we train the prediction model on the unlabeled sub-images using active learning until convergence.

The semi-supervised learning algorithm is applied for the unlabeled remaining sub-images that are not chosen during active learning [16]. This algorithm adds class central sub-images to describe the class distribution into the labeled training set, then the class boundary could be very beneficial for accelerating the convergence [33].

Class central sub-images are more likely to exist in the non-queried set of sub-images, then the updated unlabeled sub-images set *U* is computed as follows:

(2) $$U=U-x_{q}$$

where $x_{q}$ is the set of sub-images queried during active learning. The semi-supervised learning algorithm explores the central sub-images which are at a median distance to the current classification hyperplane. In case of scene rendering, the unlabeled sub-images should be classified into two classes: the noisy sub-image class denoted as *N* and the low-noisy sub-image class denoted as *R* as follows:

(3) $$N=\left\{x_{i}|x_{i}\in U,f\left(x_{i}\right)<0\right\}$$

(4) $$R=\left\{x_{i}|x_{i}\in U,f\left(x_{i}\right)>0\right\}$$

where $f(x_{i})$ is the output of the prediction model for the sub-image $x_{i}$. During the iteration of active learning, the score changing rate can be computed as follows:

(5) $$\varpi \left(x_{i}\right)=\left(\sum_{t=j}^{j+P-1}ch_{t}\left(x_{i}\right)\right)/P$$

where *P* is the number of iterations of active learning and $ch_{t}\left(x_{i}\right)$ is defined as follows:

(6) $$ch_{t}\left(x_{i}\right)=\left\{\begin{array}{cc} 0, & \mathrm{if}\;l_{t}\left(x_{i}\right)\neq l_{t-1}\left(x_{i}\right)\\ 1, & \mathrm{if}\;l_{t}\left(x_{i}\right)=l_{t-1}\left(x_{i}\right) \end{array}\right.$$

where $l_{t}\left(x_{i}\right)$ is the label of the sub-image $x_{i}$ at time t given by the model itself. The proposed semi-supervised learning algorithm computes the score changing values for the unlabeled sub-images of each class, and selects the sub-image central class of which the changing value score remains unchanged during the iterations of the active learning algorithm as follows:

(7) $$U_{N}=\left\{x_{i}|x_{i}\in N,\varpi \left(x_{i}\right)=0\right\}$$

(8) $$U_{R}=\left\{x_{i}|x_{i}\in R,\varpi \left(x_{i}\right)=0\right\}$$

Then, by using $U_{R}$ and $U_{N}$, the two sub-images that are chosen by the learning algorithm from the noisy and the low-noisy classes have the median distance to the current classification hyperplane as follows:

(9) $$x_{N}=median_{x_{i}}\left(d\left(x_{i}\right)|x_{i}\in U_{N}\right)$$

(10) $$x_{R}=median_{x_{i}}\left(d\left(x_{i}\right)|x_{i}\in U_{R}\right)$$

Then, by using the changing value score, the sub-image sets $U_{R}$ and $U_{N}$ would have a higher confidence on unlabeled sub-image predicted scores. The deep active semi-supervised learning for global illumination algorithms is given by Algorithm 1.
**Algorithm 1** Deep active semi-supervised learning algorithm**Require:***Set of scenes; P: Number of iterations of active learning; S: Number of iterations of semi-supervised learning.*1:**for all** (Scenes) **do**2:  Read-Randomly(sub-image).3:  I← CNN(sub-image).4:  **if** Labeled(sub-image) **then**5:   L←*I*6:  
**else**
7:   U←*I*8:  
**end if**
9:**end for**10:Train-Prediction-Model(*L*).11:**for**k∈{1,⋯,S}**do**12:  
**for** l∈{1,⋯,P} **do**
13:   L← Run-Active-Learning(U).14:  
**end for**
15:  Calculate-Labels-Changing-Rate(U)16:  $I_{+}$← Select-Class-Center(noisy).17:  $I_{-}$← Select-Class-Center(low-noisy).18:  $L_{+}$← Add-Predicted-Labels($I_{+}$).19:  $L_{-}$← Add-Predicted-Labels($I_{-}$).20:  L← Add($I_{+}$,$L_{+}$, $I_{-}$,$L_{-}$).21:  U← Remove($I_{+}$, $I_{-}$).22:  Retrain-Model(L).23:**end for**24:**repeat**25:  L← Run-Active-Learning(U).26:**until** Stopping Criterion

## 5. Experimental Results

### 5.1. Experimental Setup

In order to select the initial parameters of the models, we conducted experiments on the 512×512 resolution scenes. The inputs for SVM and deep SVM (DSVM) were vectors of size 16,384 (128×128) and 26, respectively. We used a cross validation with different RBF kernels on the scenes (a)–(c) to determine the standard deviation σ and the penalty factor *C* [46]. It consisted of dividing the data into *V* groups of the same size and putting a group away. The learning was then carried out on the V−1 other groups and the product model was used to predict the excluded group. In an iterative way, the same treatment was repeated on all *V* groups tests, removing each time a group. The precision estimate was the total precision rate across all *V* group tests. The learning set was split into 303 groups each of size 16 sub-images. Table 3 shows the models optimal parameters. It gives the penalty factor *C*, which controls the trade-off between complexity of the model and the number of non-separable sub-images, and the standard deviation σ for the radial basis functions that verify Mercer’s condition. The Mercer’s theorem tells us only whether a kernel is actually an inner-product kernel in some space, and therefore, admissible for use in a support vector machine. In mathematics, specifically in functional analysis, Mercer’s theorem states that a symmetric, and positive-definite matrix can be represented as a sum of a convergent sequence of product functions. From Mercer’s theorem, a matrix is a Gram Matrix if and only if it is positive and semi-definite, i.e., it is an inner product matrix in some space. For a function to be a kernel, the inner product matrix created by a data-set should necessarily be positive-semi-definite [52]. We notice that SVM needs less parameters than deep SVM and gives higher precision, which is equal to 96.24%.

Experiments were also done on the 800×800 resolution scenes. The SVM and DSVM used as input a vector of size equal, respectively, to 40,000 (200×200) and 26. The scenes (g) and (i) were used for cross-validation in order to find the optimal parameters. The set of sub-images was split into 235 and 630 groups respectively for SVM and DSVM, each of size 16 sub-images. The model optimal parameters are shown in Table 4. We find that SVM needs less number of parameters than deep SVM and gives higher precision, which is equal to 97.50%.

### 5.2. Parameter Fine-Tuning

We first considered the sub-images of the scenes with 512×512 resolution computed by the path tracing algorithm. The initial set of labeled sub-images contained only 505 sub-images selected from the scene (a). The set of unlabeled sub-images contained 9191 sub-images selected from the remaining sub-images of the scenes.

**Figure 7:**
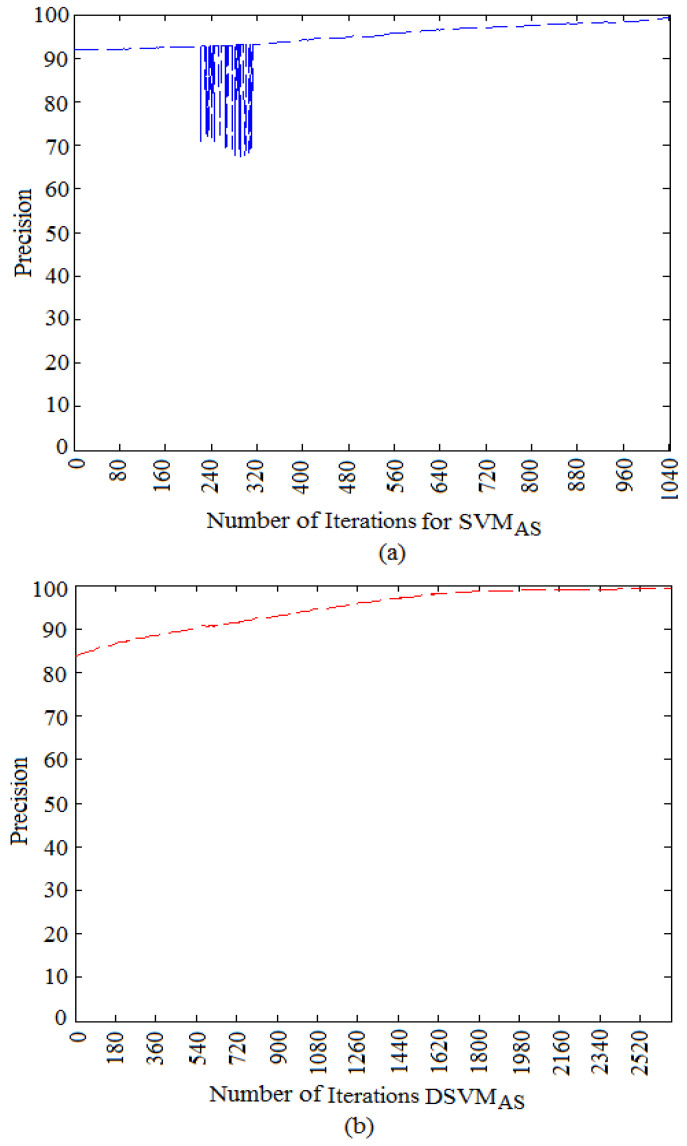
Variation of precision during the iterations of the active learning algorithm: (**a**) ${\mathrm{SVM}}_{\mathrm{AS}}$ (**b**) ${\mathrm{DSVM}}_{\mathrm{AS}}$.

We found that the performance of the active semi-supervised learning algorithm quickly increases at the first 10 iterations, so we used, S=10 and P=5 as initial values for the active semi-supervised algorithm. The active learning iterated 50 times and the semi-supervised learning added 20 sub-images centers with their predicted labels for SVM and DSVM. In each iteration, active learning took the sub-images that were closest to the current classification hyper-plane; it selected such sub-images and asked the expert for their labels. The actual contribution of the expert in the active phase was to give the active learning algorithm the correct label for each sub-image that had the lowest confidence in order to improve the performance of the model. Active learning needs human involvement and aims at selecting the most useful sub-images for training. It can greatly improve the model’s performance and can accelerate the convergence speed. During active learning, 1091 and 3212 sub-images were added to the set of labeled sub-images respectively for SVM and DSVM, in order to obtain a maximum precision equal to 99%. In order to obtain a good stopping criterion, we computed the precision on the set of the sub-images that were not selected during active learning, given that all our global illumination sub-images were labeled by human observers as shown in Section 3. In this case, the precision was equal to the percentage of sub-images classified correctly on this set. In practice, and during online decision, it should be important to define a testing set of labeled sub-images in order to compute the precision and to find a good stopping criterion. The variation of precision for SVM and DSVM active semi-supervised learning (${\mathrm{SVM}}_{\mathrm{AS}}$ and ${\mathrm{DSVM}}_{\mathrm{AS}}$) is shown in Figure 7. It is clear that, in case of ${\mathrm{SVM}}_{\mathrm{AS}}$, the precision increases after few oscillations by querying some pertinent sub-images until it reaches its maximum value after 1040 iterations. This might be due to the fact that, in this space, the two classes of sub-images overlap severely.

**Figure 8:**
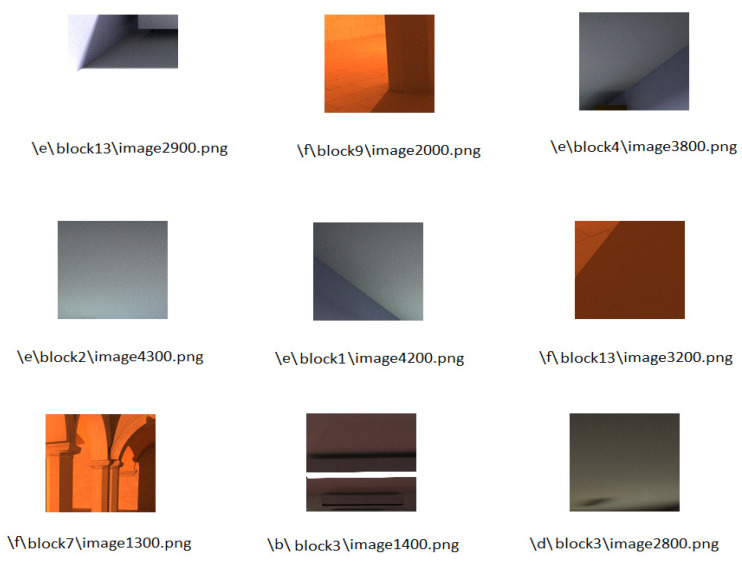
The nine sub-images added by the active learning algorithm.

Figure 8 shows the nine images added at the first iterations of the active learning algorithm. It contains, for each selected sub-image, the scene name to which it belongs, its block number, and the number of paths computed by the path tracing algorithm in order to generate it. In order to accelerate the convergence, it is shown that most of the sub-images selected are noisy and have a number of paths near the boundary of decision, which is close to the human visual score.

The number of kernels was equal to 983 and 2584 for ${\mathrm{SVM}}_{\mathrm{AS}}$ and ${\mathrm{DSVM}}_{\mathrm{AS}}$, respectively. Moreover, the ${\mathrm{DSVM}}_{\mathrm{AS}}$ model yielded a good precision with a number of parameters less than ${\mathrm{SVM}}_{\mathrm{AS}}$. The number of parameters was equal to 26×2584+2584=69,768 for ${\mathrm{DSVM}}_{\mathrm{AS}}$ and 128×128×983+983=16,106,455 for ${\mathrm{SVM}}_{\mathrm{AS}}$.

Next, we selected the sub-images of the scenes with 800×800 resolution computed by the path tracing algorithm. The initial set of labeled sub-images contained only 475 sub-images selected from the scene (i). The set of unlabeled sub-images contained 9605 sub-images selected from the remaining sub-images of the scenes. The semi-supervised learning program was executed with the initial parameters S=10 and P=5. After 50 iterations of active learning, the semi-supervised learning program added 20 sub-images centers with their predicted labels to the set of labeled sub-images. The active learning algorithm added 2386 sub-images to the set of labeled sub-images in case of ${\mathrm{SVM}}_{\mathrm{AS}}$, and 3285 in case of ${\mathrm{DSVM}}_{\mathrm{AS}}$. The maximum precision is equal to 99% and 93.4%, respectively, for ${\mathrm{SVM}}_{\mathrm{AS}}$ and ${\mathrm{DSVM}}_{\mathrm{AS}}$. The number of kernels for ${\mathrm{SVM}}_{\mathrm{AS}}$ and ${\mathrm{DSVM}}_{\mathrm{AS}}$ was equal, respectively, to 1402 and 2066.

### 5.3. Performance Comparison

#### 5.3.1. Comparative Study with SVM

In this study, we compared the active semi-supervised learning algorithm with SVM in order to show its robustness. We tuned the parameters of the SVM and DSVM models on the 3232 sub-images of the 512×512 resolution scenes (a) and (b). The optimal number of kernels was equal to 1223 and 1560, respectively, for SVM and DSVM. We noticed that DSVM used a number of parameters less than ${\mathrm{DSVM}}_{\mathrm{AS}}$, ${\mathrm{SVM}}_{\mathrm{AS}}$ and SVM. The number of parameters for this model was equal to 26×1560+1560=42,120.

**Figure 9:**
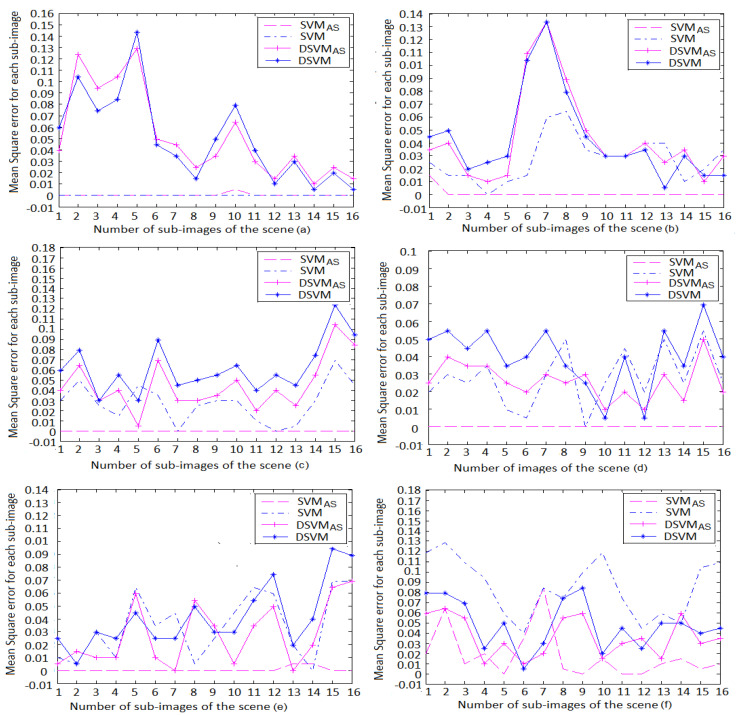
Variation of the mean square error for the 512×512 resolution scenes. It is shown that the ${\mathrm{SVM}}_{\mathrm{AS}}$ model achieves higher performance on the scenes (**b**–**f**). However, the SVM model assures higher performance than ${\mathrm{SVM}}_{\mathrm{AS}}$ on the learning scene (**a**). We also found that the ${\mathrm{DSVM}}_{\mathrm{AS}}$ model gives better performance than DSVM does on the scenes (**b**–**f**) and provides mean square errors less than SVM on the scenes (**d**–**f**).

**Figure 10:**
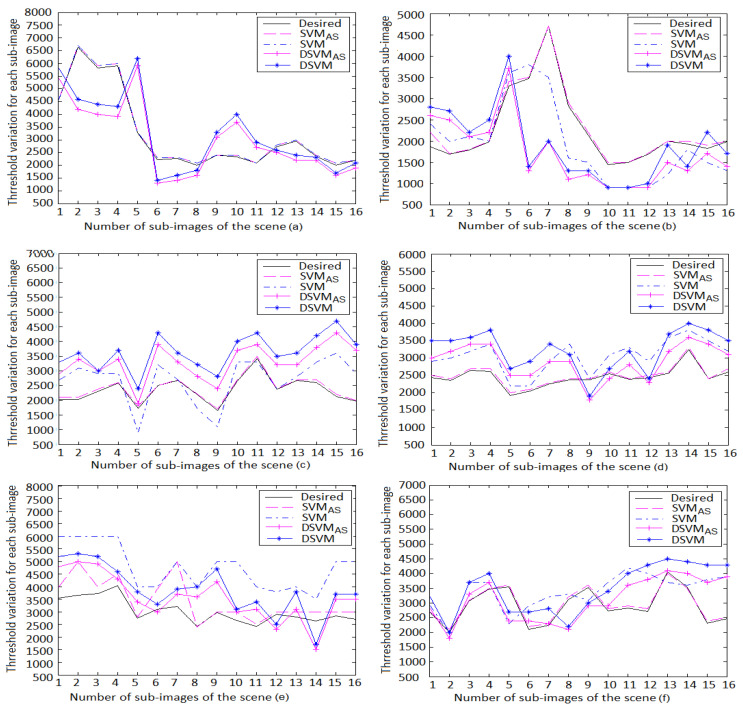
Variation of the thresholds for the 512×512 resolution scenes. It is shown that the ${\mathrm{SVM}}_{\mathrm{AS}}$ gives similar results to those of the human visual system (HVS) on the scenes (**a**–**f**). We also found that the ${\mathrm{DSVM}}_{\mathrm{AS}}$ model gives more coherent results to those of the human visual system (HVS) than DSVM does on the scenes (**a**–**f**) and achieves a better precision than SVM on the scenes (**d**–**f**).

The models’ performances were compared on all global illumination scenes. The variation of the mean square error and the mean square error ranges are listed in Figure 9 and Table 5, respectively. It is clearly seen that ${\mathrm{SVM}}_{\mathrm{AS}}$ achieves higher performance on the scenes (b)–(f). The mean square error is between 0 and 0.09 for this model, except for the blocks 6 and 7 of the scene (f), where the ${\mathrm{DSVM}}_{\mathrm{AS}}$ model assures a better performance than other models. However, the SVM model assures higher performance than ${\mathrm{SVM}}_{\mathrm{AS}}$ on the scene (a) considering that this scene is used to tune its parameters. Moreover, it is shown that ${\mathrm{DSVM}}_{\mathrm{AS}}$ model gives better precision than DSVM does on the scenes (b)–(f) and provides mean square errors less than SVM on the scenes (d)–(f). Finally, the actual thresholds of the models and the desired ones obtained by the human visual system (HVS) are also compared to show the effectiveness of our approach (Figure 10). It is shown that the ${\mathrm{SVM}}_{\mathrm{AS}}$ gives similar results to those of the human visual system (HVS) on the 512×512 resolution scenes. We also found that the ${\mathrm{DSVM}}_{\mathrm{AS}}$ model gives more coherent results to those of the human visual system (HVS) than DSVM does on the 512×512 resolution scenes and achieves a better precision than SVM on the scenes (d)–(f). In addition, it is clearly seen that ${\mathrm{SVM}}_{\mathrm{AS}}$ is better in precision than ${\mathrm{DSVM}}_{\mathrm{AS}}$. However, the ${\mathrm{DSVM}}_{\mathrm{AS}}$ model needs less parameters for fast rendering applications.

We also tested the accuracy of the models by using the $F_{1}$ score. The highest possible value of $F_{1}$ is 1, indicating perfect precision and recall [53]. Table 6 shows the variation of the $F_{1}$ score of the deep learning models for the 512×512 resolution scenes. It is shown that the ${\mathrm{SVM}}_{\mathrm{AS}}$ and ${\mathrm{DSVM}}_{\mathrm{AS}}$ models assure a better accuracy than other models.

Next, we considered the scenes with 800×800 resolution and we tuned the SVM and DSVM models on the scenes (g) and (i). The experimental learning set contained 3760 sub-images. The number of kernels for SVM and DSVM was equal, respectively, to 2367 and 1380. It was observed that the DSVM model used a number of parameters less than the other learning models. The number of parameters for this model was equal to 26×1380+1380=37,260.

**Figure 11:**
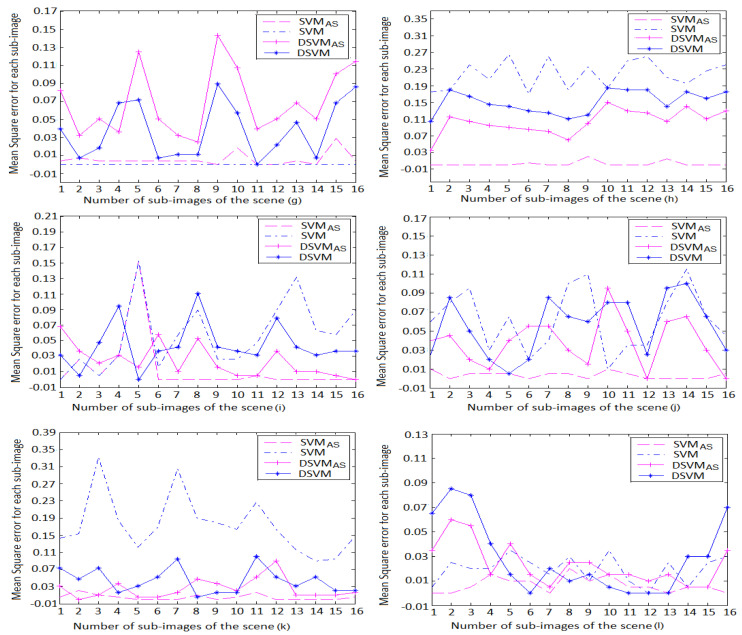
Variation of the mean square error for the sub-images of the 800×800 resolution scenes. It is shown that the ${\mathrm{SVM}}_{\mathrm{AS}}$ model gives the best performance on the scenes (**h**–**l**). We also found that ${\mathrm{DSVM}}_{\mathrm{AS}}$ provides mean square errors less than SVM and DSVM on the scenes (**h**–**l**). However, the DSVM model assures higher performance than ${\mathrm{DSVM}}_{\mathrm{AS}}$ on the learning scene (**g**).

**Figure 12:**
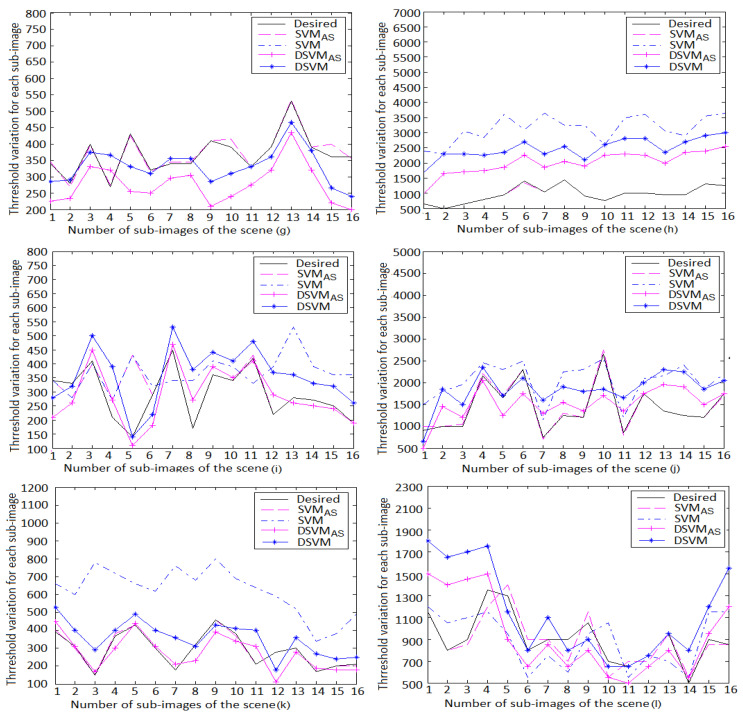
Variation of the thresholds for the sub-images of the 800×800 resolution scenes. We found that ${\mathrm{SVM}}_{\mathrm{AS}}$ gives similar results to those of the human visual system (HVS) on the scenes (**g**–**l**). It is shown that the ${\mathrm{DSVM}}_{\mathrm{AS}}$ model achieves a better precision than SVM and DSVM on the scenes (**h**–**l**).

Finally, we applied the path rendering algorithm by adding at each iteration different number of paths to each sub-images based on its noise level (Section 2). For each block of the selected scenes, the variation of the mean square error (Figure 11), the thresholds of the learning models, as well the desired human visual thresholds are shown in Figure 12.

The mean square error ranges for the learning models are listed in Table 7. We find that ${\mathrm{SVM}}_{\mathrm{AS}}$ gives the best performance on the 800×800 resolution scenes. The mean square error is between 0 and 0.03 for all scenes with the exception of the block 5 in the scene (i) where the ${\mathrm{DSVM}}_{\mathrm{AS}}$ model assures a better performance than ${\mathrm{SVM}}_{\mathrm{AS}}$ with a mean square error equal to 0.02.

It should be also noticed that ${\mathrm{DSVM}}_{\mathrm{AS}}$ provides mean square errors less than DSVM on all the 800×800 resolution scenes apart from the scene (g) that is selected to tune the DSVM model parameters. This study also shows that ${\mathrm{SVM}}_{\mathrm{AS}}$ gives similar results to those of the human visual system (HVS) on all the used scenes. However, the ${\mathrm{DSVM}}_{\mathrm{AS}}$ model uses a simple architecture for fast path tracing algorithms.

Table 8 shows the variation of the $F_{1}$ score of the deep learning models for the 800×800 resolution scenes. It is shown that the ${\mathrm{SVM}}_{\mathrm{AS}}$ and ${\mathrm{DSVM}}_{\mathrm{AS}}$ models assure a better accuracy than other models.

#### 5.3.2. Comparison Based on Memory Space and Time Complexities

We tested the time and space complexities for our models on a PC with 2 Intel CPUs at 2.54 GHZ and 16 GB of RAM using Matlab. Table 9 shows the number of parameters needed for each model and the times in seconds needed for learning and testing on any block of the 512×512 resolution scene. A comparative study based on time complexity between this model and the pooling spike neural network defined in our previous research [7] shows that the testing time for the pooling spike neural network on the same number of sub-images is equal to 0.01 s; however, it is equal to 0.008 s for the deep active semi-supervised model.

It is shown that the ${\mathrm{DSVM}}_{\mathrm{AS}}$ model is computationally efficient for real world applications because it gives a small run-time on a block of sub-images (101 sub-images).

#### 5.3.3. Comparison with Other State-of-the-Art Deep Learning Models

**Figure 13:**
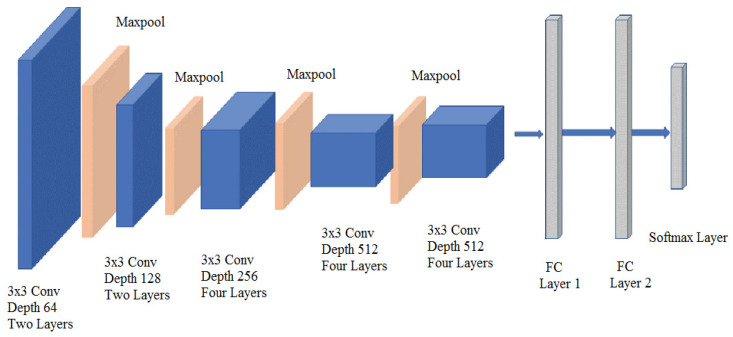
Architecture of the VGG19 Neural Network.

**Figure 14:**
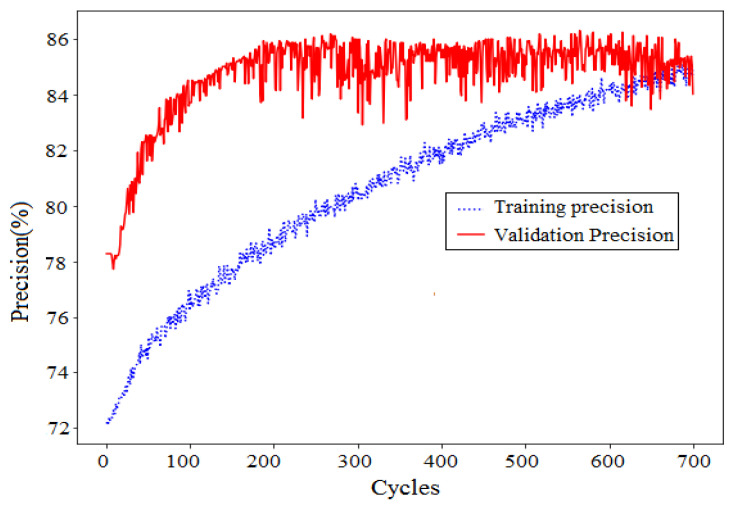
Variation of precision during the learning process of the VGG19 network (maximum precision equal to 86% after 1000 cycles).

**Figure 15:**
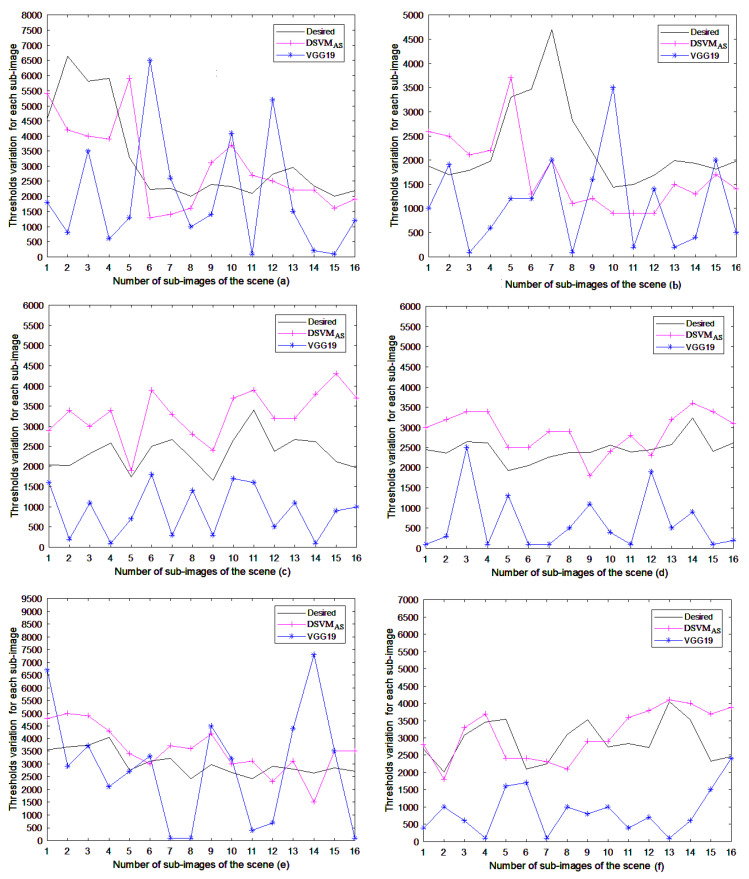
Variation of the actual thresholds of the VGG19 model, the human visual system score, and the ${\mathrm{DSVM}}_{\mathrm{AS}}$ model for the sub-images of the 512×512 resolution scenes. We found that ${\mathrm{DSVM}}_{\mathrm{AS}}$ gives more coherent results to those of the human visual system (HVS) than the VGG19 model on the scenes (**a**–**f**).

We considered first the VGG19 model which is composed of two parts. The first part, which is called the convolution base of the network, is composed of a stack of convolution and max pooling layers. The second part, which is called the classifier, is generally composed of two fully connected layers and a softmax layer. The main objective of the classifier is to classify the image according to the characteristics detected in the convolution base (Figure 13). We do transfer learning, which is a popular method in computer vision because it allows for building precise models in a considerable time saving. With transfer learning, instead of starting the learning process from scratch, deep learning models that have been learned when solving different problems are used. We first ran the convolution base of the VGG19 network over our image set composed from the scenes (a) and (c)–(f) in order to obtain good precision by using a large labeled images set to construct accurate model. Then, we used the output features of the convolution base as input to retrain the parameters of a fully connected layer with 256 sigmoid hidden units, a dropout layer, and a fully connected layer with one sigmoid output unit. The maximum precision on the evaluation scene (b) is equal to 86% after 1000 cycles (Figure 14). The learning process stopped when the generalization performance had peaked its maximum value on the evaluation scene (b). Next, we applied the path tracing algorithm on the 512×512 resolution scenes. Figure 15 shows the variation of the actual thresholds of the VGG19 model, the human visual system (HVS) score, and the ${\mathrm{DSVM}}_{\mathrm{AS}}$ model. It is shown that ${\mathrm{DSVM}}_{\mathrm{AS}}$ gives more coherent results to those of the human visual system (HVS) than the VGG19 model.

Next, we evaluated our proposed methodology against MobileNet, InceptionV3, and DenseNet201 deep learning models [54]. The scenes (a) and (c)–(f) are used for training and the scene (b) is selected for the evaluation process. Table 10 shows the maximum precision for the deep learning models on the scene (b) after 1000 training cycles. It shown that the ${\mathrm{SVM}}_{\mathrm{AS}}$ and ${\mathrm{DSVM}}_{\mathrm{AS}}$ models assure a higher precision than other state-of-the-art deep learning models.

#### 5.3.4. Comparison between Active and Semi-Supervised Learning

**Figure 16:**
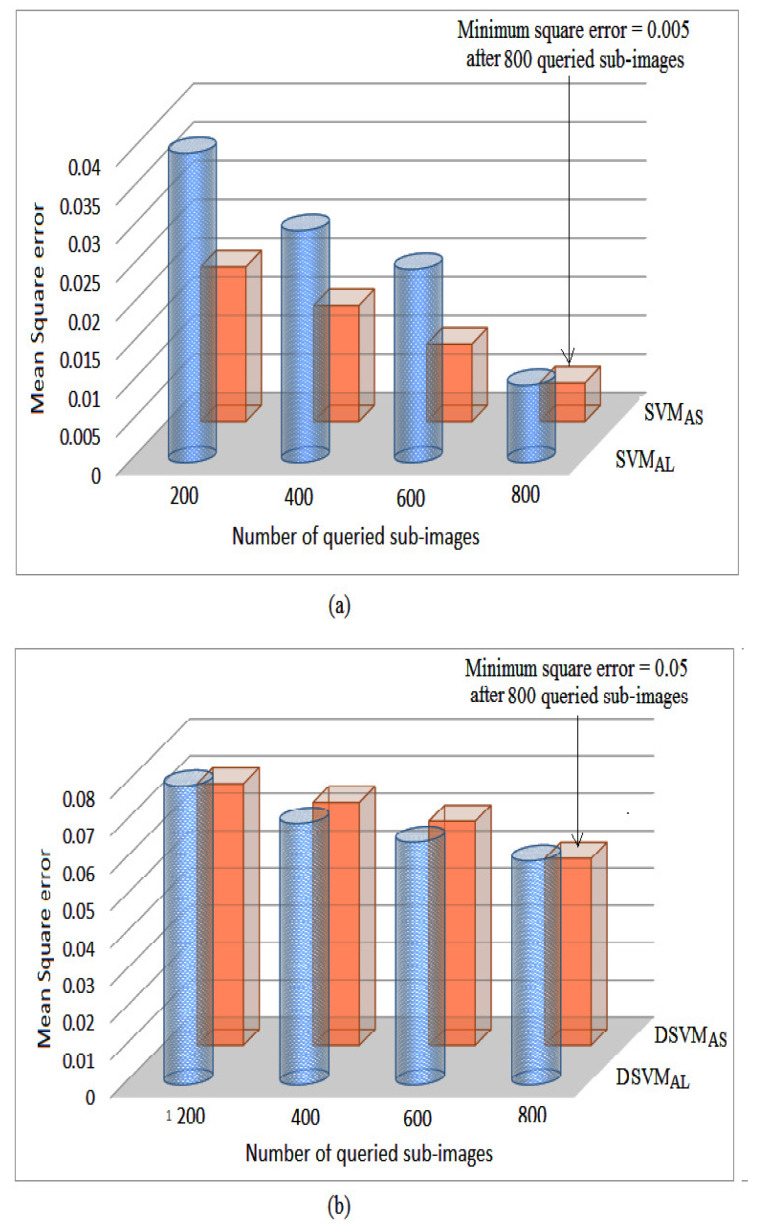
The mean square error over the number of queries when performing learning on the 512×512 resolution scenes. (**a**) ${\mathrm{SVM}}_{\mathrm{AS}}$ and ${\mathrm{SVM}}_{\mathrm{AL}}$ (**b**) ${\mathrm{DSVM}}_{\mathrm{AS}}$ and ${\mathrm{DSVM}}_{\mathrm{AL}}$.

We further compared our active semi-supervised learning algorithms ${\mathrm{SVM}}_{\mathrm{AS}}$ and ${\mathrm{DSVM}}_{\mathrm{AS}}$ with the active learning models ${\mathrm{SVM}}_{\mathrm{AL}}$ and ${\mathrm{DSVM}}_{\mathrm{AL}}$. In Figure 16a, we show the mean square errors of each scene over the number of images queried when performing learning on 512×512 resolution scenes. We find that the ${\mathrm{SVM}}_{\mathrm{AS}}$ model is better in performance than the ${\mathrm{SVM}}_{\mathrm{AL}}$ model. However, when we compared the ${\mathrm{DSVM}}_{\mathrm{AS}}$ model with ${\mathrm{DSVM}}_{\mathrm{AL}}$, similar performance was obtained (Figure 16b). We conclude that the proposed active semi-supervised algorithm provides advantageous results compared with active learning when the two classes overlap severely. However the two algorithms give similar performance otherwise.

## 6. Discussion

We have proposed a novel active semi-supervised learning method, utilizing the powerful deep approach for finding a stopping criterion, when a perceptive convergence is reached in global illumination algorithms. The active learning algorithm adds labeled data to the training set at each iteration from an imperfect images-set by a selective sampling strategy followed by queries, and is expected to improve detection performance. The approximation of an image using path tracing algorithm are widely used in computer graphics, they are prone to create imperfect shadowing effects since only the visible parts of the scene are taken into account [55].

While active learning selects the most uncertain images based on the assumption of a perfect expert, a semi-supervised algorithm adds samples by taking the unlabeled images at median distance to the current classification hyper-plane and by considering the label changing rate to ensure labeling reliability. This method achieved competitive results on global illumination scenes with different resolutions containing diffuse and specular surfaces. This method did not require a large validation set to tune the parameters of the learning model. This is considered a huge advantage over many state-of-the-art deep learning algorithms, where the dependency on large validation sets to adjust parameters is a disadvantage.

These experiments lead us to several observations that will be useful in developing in the future a detection system based on imperfect data. These methods perform well when the learning and the testing processes are initially done with a Path Tracing algorithm on standard global illumination scenes. However, rendering a single image is difficult to interpret because of image artifacts which hinder structure determination. The general solution to this problem is to generate multiple images of the same data from displaced view points and view them sequentially. In this case, multiple images of complex data involve significant allocation of both time and computing resources and the depth information measure is only available while the image is in motion. This problem can be solved by generating a single stereoscopic pair. Stereoscopy gives unambiguous depth information when it is the only available depth cue, and depth discrimination is enhanced when other cues are also included.

Only two images need to be rendered, the two halves of the stereo pair, and no motion is necessary. Images can be examined with no loss of perceived structure. In future research, we will try to accelerate global illumination rendering in case of computer generated stereoscopic images. The thresholds computed in this paper were realized in LISIC laboratory based on a training database built from experimentation performed with students. These students do not have any knowledge about computer graphics. We were therefore interested in knowing whether other people would perceive noise in the same sub-images and the difference in accuracy in their perception. Thus, experiments were performed with different users. The results we obtained show that the thresholds remain coherent with the previous ones by 95% of the users.

The results show that it is possible to achieve a detection performance that outperforms the performance obtained with fully labeled images, even when a fraction of the training data are used in the training set. As a practical matter, the experiments show that the active semi-supervised learning can be applied to an existing perception model that was originally designed for supervised training and it requires a small time during testing, so it can be used to make online decisions on photo-realistic images. Experiment results also prove that the deep active semi-supervised framework can significantly outperform supervised models for accelerating a global illumination algorithm and confirm that the proposed method can be applied on a wide variety of applications.

## 7. Conclusions

The path rendering algorithm generates theoretically correct images with an infinite number of paths. To render an image in a finite time, the number of paths has to be set before launching the rendering algorithm. Limiting the number of paths introduces a remaining variance, which can be seen as rendering noise in the final image. To analyze the quality of the final image, it would be interesting to have a noise model introduced by a rendering algorithm, and it is under investigation. However, such a model can be difficult to obtain and is specific to the considered algorithm, whereas many algorithms are implemented in modern renders. This paper introduces the application of deep active semi-supervised learning algorithm for stochastic noise detection in rendering algorithms. The introduced approach selects the pertinent sub-images using active semi-supervised learning and the stochastic noise features for the prediction models by using a specific deep convolution neural network. It is shown that the ${\mathrm{SVM}}_{\mathrm{AS}}$ model assures the best performance based on minimum square error and threshold accuracy on the different global illumination scenes; however, it needs a high questioning time which slows down its response during online rendering. It is clear that the introduced deep active semi-supervised model (${\mathrm{DSVM}}_{\mathrm{AS}}$) offers high prediction on the testing set when compared with SVM, DSVM, and VGG19 models. In this case, the performance of the model is improved by selecting only the pertinent sub-images during learning.

In addition to offering a good prediction on the testing base, this model gives a small questioning time on a block of sub-images so it is computationally efficient for online rendering in case of real world applications. Indeed, we think that the new paradigm we have introduced (specific CNN learning using active semi-supervised algorithm) should be of great interest for a great number of knowledge-base models that are involved in numerous applications.

In our case, a future research method could be to use deep active semi-supervised learning in case of computer generated stereoscopic images, since convolution neural network is less sensitive to the curse of dimensionality. Our technique can also be compared with other approaches such as deep spike neural networks in case of higher dimension images (HDR images). It is also important to investigate how to optimize our work on a GPU card using parallel computing. Finally, our approach also requires building a larger number of images set computed with different rendering techniques.

## Figures and Tables

**Table 1:** Number of paths per pixel between two successive sub-images and largest number of paths per pixel for the scenes with 800×800 resolution.

Scenes with 800×800 Resolution	Number of Paths between Two Successive Sub-Images	Largest Number of Paths
(g)	5	700
(h)	50	5000
(i)	10	950
(j)	50	5000
(k)	10	950
(l)	50	5000

**Table 2:** Deep architecture.

Layers	C1	C2	C3	C4	C5	C6	C7	C8	C9	C10	C11	C12
**Size**	3×3	5×5	3×3	5×5	3×3	5×5	3×3	5×5	3×3	5×5	3×3	5×5
**Padding**	1	2	1	2	1	2	1	2	1	2	1	2

**Table 3:** Optimal parameters for the models for the scenes with 512×512 resolution. The bold value simply indicates the maximum precision.

Learning Model	Penality Factor C	Standard Deviation	Precision	Mean Number of Kernels
SVM	8	50	**96.24**%	1750
DSVM	8	70	90.7%	1970

**Table 4:** Optimal parameters for the models for the 800×800 resolution scenes. The bold value simply indicates the maximum precision.

Learning Model	Penality Factor C	Standard Deviation	Precision	Mean Number of Kernels
SVM	8	70	**97.50**%	1770
DSVM	8	120	83.22%	5350

**Table 5:** Mean square error range for the 512×512 resolution scenes. The bold values simply indicate the minimum Mean square error range.

Scenes	SVM	${\mathrm{SVM}}_{\mathrm{AS}}$	DSVM	${\mathrm{DSVM}}_{\mathrm{AS}}$
(a)	0	**[0–0.01]**	[0–0.15]	[0–0.13]
(b)	[0–0.07]	**[0–0.02]**	[0–0.14]	[0–0.14]
(c)	[0–0.07]	**0**	[0–0.13]	[0–0.11]
(d)	[0–0.06]	**0**	[0–0.07]	[0–0.05]
(e)	[0–0.07]	**[0–0.01]**	[0–0.1]	[0-0.07]
(f)	[0.04–0.13]	**[0–0.09]**	[0–0.09]	**[0–0.07]**

**Table 6:** $F_{1}$ score for the 512×512 resolution scenes. The bold values simply indicate the maximum $F_{1}$ score.

Scenes	SVM	${\mathrm{SVM}}_{\mathrm{AS}}$	DSVM	${\mathrm{DSVM}}_{\mathrm{AS}}$
(a)	1	**0.99**	0.92	**0.93**
(b)	0.95	**0.99**	0.94	**0.95**
(c)	0.95	**1**	0.91	**0.95**
(d)	0.96	**1**	0.94	**0.97**
(e)	0.95	**0.99**	0.94	**0.96**
(f)	0.86	**0.97**	0.92	**0.95**

**Table 7:** Mean square error range on the 800×800 resolution scenes. The bold values simply indicate the minimum Mean square error range.

Scenes	SVM	${\mathrm{SVM}}_{\mathrm{AS}}$	DSVM	${\mathrm{DSVM}}_{\mathrm{AS}}$
(g)	0	**[0–0.03]**	[0–0.09]	[0.01–0.15]
(h)	[0.15–0.27]	**[0–0.03]**	[0.07–0.19]	[0.03–0.15]
(i)	[0–0.15]	[0–0.15]	[0–0.13]	**[0–0.07]**
(j)	[0.05–0.13]	**[0–0.01]**	[0-0.11]	[0–0.11]
(k)	[0.07–0.35]	**[0–0.03]**	[0-0.11]	[0–0.11]
(l)	[0–0.05]	**[0–0.03]**	[0–0.09]	[0–0.05]

**Table 8:** $F_{1}$ score for the 800×800 resolution scenes. The bold values simply indicate the maximum $F_{1}$ score.

Scenes	SVM	${\mathrm{SVM}}_{\mathrm{AS}}$	DSVM	${\mathrm{DSVM}}_{\mathrm{AS}}$
(g)	1	**0.99**	0.90	**0.90**
(h)	0.63	**0.99**	0.77	**0.85**
(i)	0.91	**0.98**	0.93	**0.97**
(j)	0.90	**0.99**	0.91	**0.95**
(k)	0.67	**0.99**	0.93	**0.96**
(l)	0.97	**0.99**	0.96	**0.97**

**Table 9:** Number of parameters and the times in seconds needed for learning and testing on the 512×512 resolution scenes. The bold values simply indicate the minimum values obtained by the models.

Model	No. of Parameters	Learning Time (s)	Testing Time (s)
SVM	20,038,855	4	0.17
DSVM	**42,120**	**1**	**0.008**
${\mathrm{SVM}}_{\mathrm{AS}}$	16,106,455	8053	0.15
${\mathrm{DSVM}}_{\mathrm{AS}}$	**69,768**	**2095**	**0.008**

**Table 10:** Maximum precision for the deep learning models after 1000 training cycles. The bold values simply indicate the higher precision.

Model	Precision (%)
InceptionV3	76
MobileNet	75
Resnet210	81
VGG19	86
${\mathrm{SVM}}_{\mathrm{AS}}$	**99**
${\mathrm{DSVM}}_{\mathrm{AS}}$	**91**

## Data Availability

The data that support the findings of this study are available from the LISIC Laboratory at ULCO University France but restrictions apply to the availability of these data, which were used under license for the current study, and so are not publicly available. Data are however available from the authors upon reasonable request and with permission of the LISIC Laboratory.
